# Transcriptional control of vertebrate neurogenesis by the proneural factor Ascl1

**DOI:** 10.3389/fncel.2014.00412

**Published:** 2014-12-02

**Authors:** Francisca F. Vasconcelos, Diogo S. Castro

**Affiliations:** Molecular Neurobiology, Instituto Gulbenkian de CiênciaOeiras, Portugal

**Keywords:** Ascl1/Mash1, neurogenesis, proneural gene, transcription, Notch signaling

## Abstract

Proneural transcription factors (TFs) such as Ascl1 function as master regulators of neurogenesis in vertebrates, being both necessary and sufficient for the activation of a full program of neuronal differentiation. Novel insights into the dynamics of Ascl1 expression at the cellular level, combined with the progressive characterization of its transcriptional program, have expanded the classical view of Ascl1 as a differentiation factor in neurogenesis. These advances resulted in a new model, whereby Ascl1 promotes sequentially the proliferation and differentiation of neural/stem progenitor cells. The multiple activities of Ascl1 are associated with the activation of distinct direct targets at progressive stages along the neuronal lineage. How this temporal pattern is established is poorly understood. Two modes of Ascl1 expression recently described (oscillatory *vs*. sustained) are likely to be of importance, together with additional mechanistic determinants such as the chromatin landscape and other transcriptional pathways. Here we revise these latest findings, and discuss their implications to the gene regulatory functions of Ascl1 during neurogenesis.

## Introduction

Neurogenesis in the developing mammalian brain is a highly complex process that requires neural progenitor cells to progress through a succession of distinct cellular states. These developmental steps have been particularly well defined in the embryonic telencephalon, where distinct types of progenitors have been identified during the neurogenesis period (Kriegstein and Alvarez-Buylla, 2009). Radial glial (RG) cells in the ventricular zone (VZ) have characteristic features of neural stem/progenitor cells, as they self-renew by asymmetric division and have the potential to differentiate into both neurons and glial cells (Götz and Huttner, 2005). Upon cell division, RG cells give rise to another RG cell and either a post-mitotic neuron, or an intermediate progenitor that can divide further to amplify the lineage (Haubensak et al., 2004; Miyata et al., 2004; Noctor et al., 2004; Pilz et al., 2013). These various progenitor types are differentially segregated between two germinal layers. Most RG cells divide at the apical surface of the VZ, while most intermediate progenitors divide more basally in the sub-ventricular zone (SVZ).

Proneural transcription factors (TFs) of the bHLH family including Ascl1 (also called Mash1) and members of the Neurogenin family are the main regulators of vertebrate neurogenesis. Both gain and loss-of-function analyses have shown they are both required and sufficient to induce a complete program of neuronal differentiation (Bertrand et al., 2002; Wilkinson et al., 2013). While genetic ablation of Ascl1 in mice results in neural developmental defects associated with reduced generation of neurons (Casarosa et al., 1999; Horton et al., 1999; Marin et al., 2000), overexpression of this TF in neural progenitors induces cell-cycle exit and full neuronal differentiation and specification (Nakada et al., 2004; Castro et al., 2006; Berninger et al., 2007b; Geoffroy et al., 2009). In line with its master regulatory role in the neuronal lineage, recent studies have revealed the ability of Ascl1 to convert various non-neural somatic cells (e.g., fibroblasts) into induced neurons (Berninger et al., 2007a; Vierbuchen et al., 2010; Karow et al., 2012), renewing interest in understanding how this important TF works at the molecular level.

## Proneural factors and the notch signaling pathway

While driving neuronal differentiation, proneural factors also activate the Notch signaling pathway in neighboring progenitors, a process that is highly reminiscent of the lateral inhibition model proposed for *Drosophila* neurogenesis (Louvi and Artavanis-Tsakonas, 2006). Proneural factors directly activate the transcription of Notch ligands such as Dll1 (Castro et al., 2006; Henke et al., 2009), which interact with a transmembrane Notch receptor in neighboring cells. This event results in the cleavage and release of the Notch intracellular domain (NICD) from the cell membrane into the nucleus, where it forms a complex with the DNA-binding TF Rbpj and additional coactivators. Direct targets of this complex include the bHLH transcriptional repressors Hes1 and Hes5, which in turn bind to the promoters of proneural genes, repressing their expression and thereby inhibiting neuronal differentiation (Kageyama et al., 2005). Thus, proneural genes are both regulators and regulated by the Notch signaling pathway, a network that functions in parallel to the differentiation program to keep—even if only transiently—adjacent cells undifferentiated. Such lateral inhibition results in proneural factors being expressed in a “salt-and-pepper” pattern and prevents simultaneous differentiation of all progenitors, ensuring that an appropriate number is maintained during embryonic development.

## A revised view of lateral inhibition in vertebrates

It is known that in a variety of cell types (e.g., fibroblasts), Hes1 expression levels regularly alternate over time due to its ability to behave as an intrinsic oscillator (Hirata et al., 2002; Masamizu et al., 2006; Kobayashi et al., 2009). Hes1 represses its own promoter in a feedback mechanism, which associated with short-lived Hes1 transcript and protein, results in autonomous oscillations of its expression with a 2–3 h period. It was recently shown that Hes1 also oscillates in neural progenitors (Shimojo et al., 2008). Because Hes1 and proneural factors display complementary patterns of expression, one possibility is that oscillation of Hes1 results in the oscillation of proneural genes. This is indeed the case for both Neurog2 and Ascl1, as shown by a variety of approaches (Shimojo et al., 2008; Imayoshi et al., 2013). Most notably was the generation of transgenic mice bearing a bacterial artificial chromosome (BAC) containing the Ascl1 regulatory regions driving the expression of Ascl1 fused to either luciferase or green fluorescent protein (GFP), where the activity of the reporter monitors faithfully the expression of the endogenous Ascl1 protein (Imayoshi et al., 2013). Proneural proteins are direct activators of Dll1, resulting in its oscillation and mutual activation of Notch signaling in neighboring progenitors (Castro et al., 2006; Shimojo et al., 2008). There is evidence that Hes1 activity both promotes and inhibits the cell cycle and therefore its oscillation may be required for cell proliferation (Castella et al., 2000; Hartman et al., 2004; Sang et al., 2008). Oscillatory expression of TFs with lineage specification functions has also been observed in other systems, and the function of such oscillations is still a matter of debate. As opposed to steady-state mode, oscillatory expression may generate heterogeneity of response of an apparently homogeneous progenitor cell population to a given input signal. In addition, different inputs may affect different parameters of the oscillation (e.g., period, amplitude) and, hence, trigger different functional outcomes (Mengel et al., 2010; Pina et al., 2012; Sequerra et al., 2013).

**Figure 1:**
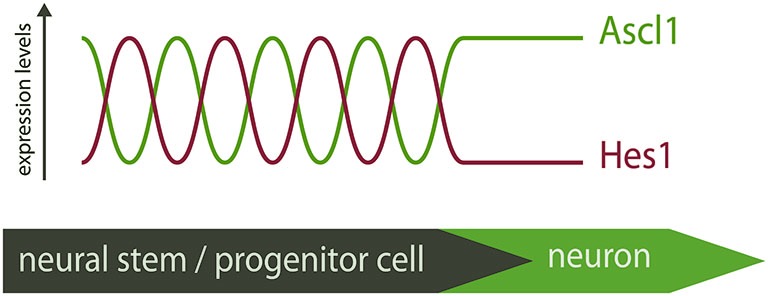
**Distinct modes of expression of Ascl1 in neural stem/progenitor cells**. Hes1 and Ascl1 oscillate in neural progenitors with a 2–3 h period. The inverse correlation of expression of Hes1 and Ascl1 proteins suggest these TFs oscillate out-of-phase. At the onset of differentiation, Hes1 expression is extinguished and Ascl1 expression becomes sustained. The “salt-and-pepper” expression of Ascl1 in the neural tube is therefore the result of a dynamic pattern of expression, and Ascl1 expression is not necessarily an indicative of differentiation.

In light of these recent findings, the “salt-and-pepper” expression pattern of proneural factors is perceived as a snapshot of a dynamic mode of expression. Proneural factors are therefore expressed in neural progenitors at different stages of differentiation, and not only in committed progenitors that will soon become post-mitotic, as was previously thought (Figure 1).

## Oscillatory vs. sustained mode of expression

Time-lapse imaging of individual neural progenitors in culture revealed that during neuronal induction, Ascl1 and Neurog2 switch from an oscillatory to a sustained mode of expression after the last cell division, followed by the expression of the neuronal marker doublecortin 6–8 h later (Shimojo et al., 2008; Imayoshi et al., 2013). This suggested the ability of proneural factors to trigger differentiation may require their expression to be sustained. Such a causal link was established upon the use of an optogenetic approach where Ascl1 expression is regulated by a light-switchable transactivator (Imayoshi et al., 2013). This system was introduced in an Ascl1 null background to investigate the consequence of different dynamics of Ascl1 expression induced by different pulses of light. An oscillatory mode with a 3 h periodicity increased proliferation, compensating the lower proliferation rate observed in Ascl1 null progenitors in culture. By contrast, sustained expression of Ascl1 for 6 h resulted in cell-cycle exit and neuronal differentiation. The same approach used in slice cultures of the dorsal telencephalon where Ascl1 is usually expressed at very low levels reached similar conclusions. What determines the transition to a sustained mode of Ascl1 expression remains an open question, but it was suggested that varying levels of NICD may play a role in this step (Imayoshi et al., 2013).

Overall, these findings explain why most evidence based on Ascl1 gain-of-function (sustained expression) points to a role in promoting differentiation (with concomitant cell cycle-exit) of progenitors (Nakada et al., 2004; Castro et al., 2006; Geoffroy et al., 2009). By contrast, knock-down of Ascl1 levels upon expression of sequence-specific shRNA decreased proliferation of neural progenitors in culture (Castro et al., 2011), while acute knock-out of Ascl1 in the ventral telencephalon caused premature cell-cycle withdrawal of both VZ and SVZ progenitors (Castro et al., 2011; Pilz et al., 2013), suggesting a role in maintaining proliferation in both RG cells and intermediate progenitors.

## Characterization of Ascl1 target genes

Proneural factors function primarily as transcriptional activators, binding in heterodimeric complexes with bHLH E-proteins to the regulatory regions of their target genes (Bertrand et al., 2002). A major leap forward in our understanding of the molecular mechanisms underlying Ascl1 function has been the progressive characterization of its transcriptional program. The advent of genomic approaches based on chromatin immunoprecipitation allowed the characterization of a large number of genes directly controlled by Ascl1 in a neurogenesis context. Two studies have used chromatin immunoprecipitation followed by hybridization to DNA arrays (ChIP-chip), or massive parallel sequencing (ChIP-seq), to characterize the Ascl1 transcriptional program in ventral telencephalon and dorsal spinal cord of the developing mouse embryo, respectively (Castro et al., 2011; Borromeo et al., 2014). A common theme of both studies was the diversity of biological functions of Ascl1 target genes, indicating Ascl1 directly controls various stages of neurogenesis, including neuronal differentiation, migration, axon guidance and synapse formation. In the ventral telencephalon, the region of the murine brain with the largest SVZ during the neurogenic period, the pro-proliferation activity of Ascl1 extends beyond the maintenance of Notch/Hes1 oscillations through activation of Dll1, and includes the activation of genes required for cell-cycle progression such as E2F1 and Foxm1 (Castro et al., 2011).

**Figure 2:**
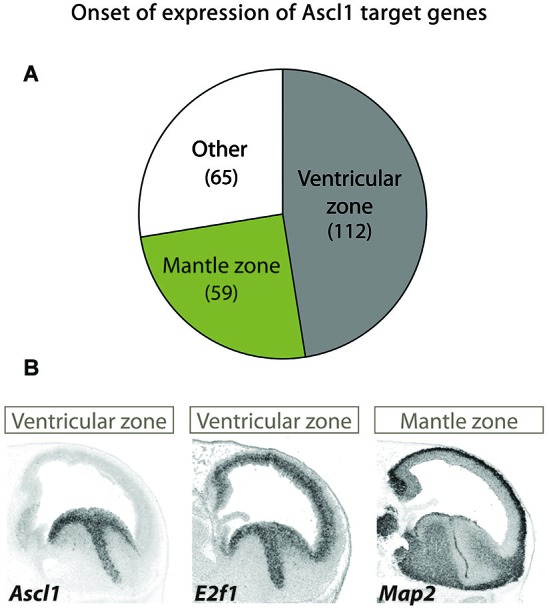
**Different patterns of expression of Ascl1 target suggest distinct kinetics of gene activation. (A)** A large screening of expression patterns of Ascl1 direct targets in embryonic mouse ventral telencephalon revealed distinct patterns of expression (Castro et al., 2011). Three distinct groups can be identified. Genes in the largest set display an onset of expression at the VZ (which includes mostly neural stem cells), while in another the onset occurs at the mantle zone (here defined as including SVZ and all other outer layers, comprising intermediate progenitors and post-mitotic neurons). A third group includes genes with a uniform or more complex pattern of expression. Number of genes in each set is indicated in between brackets. **(B)** Examples of two Ascl1 direct targets with distinct patterns of expression revealed by *in situ* hybridization on sagittal sections of mouse telencephalon at E13.5 stage of embryonic development. Expression of the proliferation gene E2f1 in VZ/SVZ mirrors that of Ascl1. By contrast, the neuronal differentiation gene MAP2 is expressed in post-mitotic neurons. Images are from Allen Developing Mouse Brain Atlas.^1^

The Ascl1 targets are associated with progressive functions along neurogenesis and have distinct onsets of expression along the neuronal lineage in this brain region, as indicated by their expression patterns. The expression of the largest group mirrors that of Ascl1 itself in both germinal layers and includes genes expected to promote cell proliferation (e.g., E2F1), whereas that of a smaller but significant group of targets is restricted to the mantle zone (e.g., MAP2) (Figure 2).

All the above observations resulted in a model whereby Ascl1 sequentially promotes proliferation and differentiation of progenitors along the neuronal lineage, with the concomitant activation of partially distinct transcriptional programs. This reconciles the classical view of Ascl1 as a differentiation factor with the fact that this proneural factor is expressed mostly in cycling cells. The different kinetics of Ascl1 targets may result from the integration of various mechanistic determinants, including its two modes of expression as briefly discussed below.

## Regulation of Ascl1 protein levels and transcriptional activity

One simple possibility is that increasing levels of Ascl1 activity, resulting from changes in the Ascl1 mode of expression (oscillatory *vs*. sustained), protein levels and/or its transcriptional function, will result in the sequential activation of promoters with an increasing threshold of response. Although not formally demonstrated, it is likely that some Ascl1 targets respond differently to the oscillatory or sustained expression of Ascl1. One possible model invokes the function of a feed-forward-loop (FFL), a motif highly enriched in transcriptional networks. A variant of such motif called coherent FFL (whereby one TF activates another TF, and both co-activate target genes) has been shown to allow for a discriminated response of the target gene triggered by transient or sustained input signals from the first TF (Shen-Orr et al., 2002; Mangan and Alon, 2003). Considering the large number of TFs found amongst Ascl1 targets, it would be of interest to investigate if any may establish with this proneural factor such a network motif, providing a mechanistic basis for differential activation of Ascl1 targets upon its two modes of expression.

Some observations suggest Ascl1 protein levels may also play a role at the onset of neuronal differentiation. Time-lapse imaging of Ascl1/luciferase expressing neural progenitors in culture revealed an increase in Ascl1 levels in 90% of the cells that undergo asymmetrical (neurogenic) cell division, against 30% of the cells that undergo a proliferative symmetric division (Imayoshi et al., 2013). Thus, although not being strictly required or sufficient, an increase in Ascl1 protein levels before cell division does bias cells towards the neuronal fate.

A few signaling pathways have been implicated in the regulation of Ascl1 protein levels in different cellular contexts (Sriuranpong et al., 2002; Viñals et al., 2004; Oishi et al., 2009). In the most striking example, varying Ascl1 protein levels regulated by retinoic acid, result in the generation of different types of neurons at the p3 domain of hindbrain and spinal cord (Jacob et al., 2013). In all cases studied however, it is unclear if and how Ascl1 levels affect the activation of its target genes. Thus, while all these results indicate that various pathways converge to control Ascl1 protein levels, their relevance to the differential regulation of subsets of Ascl1 targets remains to be explored.

Two recent studies provided evidence that Ascl1 function can be modulated by phosphorylation at multiple serine-proline sites. During cortical development, manipulating RAS/ERK signaling to abnormal high level induces a Neurog2 to Ascl1 switch of expression and modifies Ascl1 activity by direct phosphorylation. As a result, Ascl1 drives a glioblast-like differentiation program reminiscent of its function in oligodendrogenesis. Another work showed that Ascl1 phosphorylation is sensitive to levels of Cdk/Cdk inhibitors, and diminishes its ability to drive primary neurogenesis in *Xenopus* embryos, providing a direct link between the cell-cycle machinery and regulation of neurogenesis (Ali et al., 2014). It is currently not known how phosphorylation impacts the neurogenic activity of Ascl1. Possible mechanisms include differential binding to DNA (as shown with Neurog2 phosphorylation) (Ali et al., 2011), or co-factor recruitment. Strikingly, phosphorylation affects the ability of Ascl1 to up-regulate the late/differentiation targets Myt1 and neural β-tubulin, while having little effect on *Dll1* induction (Ali et al., 2014). Moreover, a differential effect is also observed on the ability of Ascl1 to transactivate the promoters of various target genes (Li et al., 2014). Differential sensitivity of promoters to Ascl1 phosphorylation may thus be one important mechanism determining which targets Ascl1 regulates in proliferating vs. differentiating progenitors.

## Importance of chromatin landscape and Ascl1 binding sites

Distinct thresholds of response to Ascl1 may result from differences in requirements for chromatin remodeling across its target genes. Very little is known however, on the impact that Ascl1 and proneural factors in general may have on the chromatin landscape when regulating gene transcription. Expression of a dominant negative form of Brg1, a catalytic component of SWI/SNF chromatin remodeling complex, blocks neuronal differentiation of P19 cells mediated by Neurog3 (Seo et al., 2005). In addition, it inhibits Neurog3 activation of the promoter of *NeuroD2*, the paradigm of a late/differentiation target of Neurogenins. In spite of this suggestive example, the importance of chromatin remodeling to the overall temporal patterning of the transcriptional program downstream of proneural factors remains to be investigated.

The chromatin landscape can contribute to restrict the accessibility of a TF to its target sites. A study of Ascl1 mediated neuronal reprogramming has recently shown that Ascl1 binds to its *bona fide* target sites when ectopically expressed in fibroblasts, even to those located within closed chromatin context, as defined by FAIRE-seq (Wapinski et al., 2013). The term “on target” pioneer factor was coined to indicate the ability of Ascl1 to recognize its cognate binding sites when ectopically expressed, as opposed to other TFs in iPS reprogramming (Soufi et al., 2012). Although the ability to bind nucleosomal DNA may argue against a dominant role of the chromatin structure in controlling Ascl1 function, it remains possible that Ascl1 accessibility to its target sites may be different in proliferating vs. differentiating progenitors.

The affinity of a TF binding site, determined by the DNA sequence, can dictate the kinetics of response of its direct targets. A striking example of how such mechanism can establish the temporal pattern of a developmental program is the activation of pharyngeal genes by the forkhead factor PHA4 at different developmental stages in *Caenorhabditis elegans* (Gaudet and Mango, 2002). Binding site mutations result in abnormalities in the timing of target gene expression *in vivo*, according to the resulting affinity to PHA4 binding. Concerning bHLH TFs, and in addition to the two central bases of the E-box, which provide specificity to distinct factors, residues flanking the hexamer sequence contribute to modulate binding affinity (Blackwell and Weintraub, 1990; Fisher et al., 1993). A study investigating the regulation of the Dll1 gene by Ascl1 has shown that residues at each side and immediately adjacent to the CAGSTG E-box determine binding affinity of Ascl1 *in vitro*, and strength of response in transcriptional assays (Castro et al., 2006). Although a possibility, the contribution of varying affinities for its binding sites to the kinetics of Ascl1 targets remains to be investigated.

## Functional interactions with other transcriptional networks

Transcriptional programs are at the intersection of multiple transcriptional networks. Thus, a comprehensive view of the dynamics of the Ascl1 program will require its integration within other transcriptional pathways operating in neural progenitors. Very few studies have so far identified functional interactions between Ascl1 and other TFs. The forkhead factor FOXO3 regulates neural stem cell maintenance and is required to preserve the neural stem cell pool in adult mice (Renault et al., 2009). Recently, it has been shown that FOXO3 inhibits Ascl1-induced neuronal differentiation in cultured neural progenitors and direct neuronal conversion in fibroblasts (Webb et al., 2013). Although the molecular basis for this antagonism is not yet clear, it is likely to make use of the large number of regulatory regions co-bound by both TFs, many of which regulate Notch pathway related genes. Also SOX1B factors (Sox1/2/3) have been previously shown to counteract proneural proteins in gain-of-function experiments in the chick neural tube (Bylund et al., 2003). Thus, the intertwining of transcriptional networks regulating neural progenitor maintenance and differentiation may be a recurrent feature to be explored in future studies.

One immediate example is the Notch pathway. Within the large number of Ascl1 targets identified in ventral telencephalon, the Rbpj consensus binding sequence is enriched at the vicinity of Ascl1 binding sites, specifically in targets that promote proliferation (Castro et al., 2011). Previous studies of neurogenesis in *Drosophila* provide important clues on how the two factors may interact at the molecular level (Nellesen et al., 1999; Cave et al., 2005). In co-bound regulatory regions with a specific cis-architecture, efficient transactivation is only achieved upon the simultaneous activation of both proneural and Notch pathways. A similar synergy between Ascl1 and Rbpj can be observed in transcriptional assays in murine cells (Cave et al., 2005), although it remains to be shown whether such interaction does take place in gene regulatory regions. RG cells can be distinguished from intermediate progenitors in ventral telencephalon based on their high levels of canonical Notch signaling (Mizutani et al., 2007), thus in principle such a mechanism could be used to differentially activate Ascl1 targets in the two types of progenitors.

## Perspective

Proneural TFs such as Ascl1 have been seen as master regulators of the neuronal lineage that play an important regulatory role at the onset of differentiation. Recent findings have uncovered a far more complex picture in which Ascl1 plays sequential functions in proliferating and differentiating neural stem/progenitor cells, with the concomitant regulation of distinct target genes. How sub-sets of the Ascl1 transcriptional program are differentially activated along the neuronal lineage is poorly understood, and will certainly result from the combination of distinct mechanistic determinants. This important question will surely remain a subject of intense research for the foreseeable future.

## Conflict of interest statement

The authors declare that the research was conducted in the absence of any commercial or financial relationships that could be construed as a potential conflict of interest.

## Footnotes

^1^Available at: http://developingmouse.brain-map.org
